# Probing the Nanostructure and Reactivity of Epoxy–Amine Interphases

**DOI:** 10.1021/acsami.4c17387

**Published:** 2024-12-09

**Authors:** Suzanne Morsch, Yanwen Liu, Kieran Harris, Flor R. Siperstein, Claudio Di Lullo, Peter Visser, Stuart Lyon

**Affiliations:** † Corrosion@Manchester, Department of Materials, The University of Manchester, Nancy Rothwell Building, Oxford Road, Manchester M13 9PL, U.K.; ‡ Department of Chemical Engineering, The University of Manchester, Nancy Rothwell Building, Oxford Road, Manchester M13 9PL, U.K.; § AkzoNobel Powder Coatings, Stoneygate Lane, Felling, Gateshead, Tyne & Wear NE10 0JY, U.K.; ∥ AkzoNobel, Rijksstraatweg 31, 2171 AJ Sassenheim, The Netherlands

**Keywords:** epoxy, interphase, AFM-IR, coating, composite, molecular dynamics simulation

## Abstract

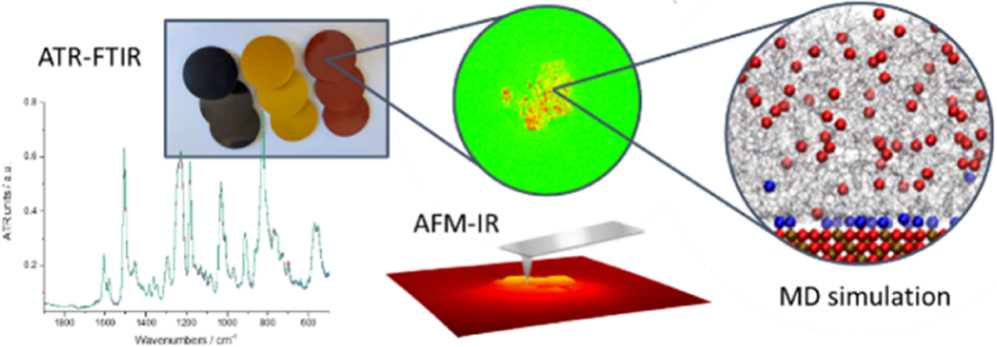

Understanding and controlling the structure of interphase regions in epoxy resins have been a long-standing goal in high-performance composite and coating development, since these are widely considered to be weak points in the microstructure of these materials, determining key properties such as fracture strength and barrier performance. These buried nanoscale regions are, however, inaccessible to conventional analytical techniques, and little is understood about their underlying formation mechanism. Here, we combine molecular dynamics (MD) simulation with nanoscale infrared chemical mapping to develop new understanding of the interphase using model epoxy–amine binders composed of diglycidyl ether of bisphenol A (DGEBA) cross-linked using *m*-xylylenediamine (MXDA). Iron oxide powders are used as exemplary surfaces, where we demonstrate that the electrostatic binding energies between the amine cross-linker and particles range from repulsive (magnetite, Fe_3_O_4_) to weakly attractive (hematite, Fe_2_O_3_) to strong immobilization (goethite, FeOOH). We find that interfacial binding occurs upon mixing and determines the overall level of residual amine content in the bulk matrix but does not correlate with a detectable amine depletion in the vicinity of particles. In all cases, an excess of both epoxy and amine functionality is detected close to particles, and the extent of matrix undercuring is found to be dependent on the entropic segregation of the unreacted material during the ambient cure. Detailed MD simulations demonstrate that spatial segregation of the unreacted precursors is expected in the interphase, leading to the experimental observation that, even after extensive postcure heating, individual particles remain embedded in a nanoscale underdeveloped environment.

## Introduction

In protective coatings, a continuous, highly cross-linked polymer matrix provides both structural integrity and a barrier to aggressive species. Homogeneous protective coating systems are commonly filled with mineral pigments, providing additional barrier properties and active corrosion inhibition and serving as fillers. As pigmented coatings cure, it is now understood that the development of the organic network is perturbed around these embedded particles, resulting in the formation of structurally distinct polymeric “interphase” regions, which are widely considered to be weak points in the microstructure of protective coatings; vulnerable interphase regions have been linked to adhesion loss, fracture toughness, and small molecule transport.^1−5^ Unfortunately, however, since the formation mechanism, nature, and extent of interphase regions remain theoretically and experimentally unresolved, formulators cannot at present control its structure. This is in large part due to the inaccessible nature of these buried nanoscale regions.

Recently, we demonstrated that detailed analysis of nanocomposites, i.e., specimens containing large areas of metal-oxide interface, offers a promising alternative to the use of thin films in interphase studies, bypassing well-known issues such as precursor volatility and the addition of excessive solvent associated with thin-film preparation.^5^ Using the nanocomposite approach, we demonstrated that minor fluctuations in the cure degree can be detected using conventional ATR-FTIR of the bulk material, as a function of added particle volume.^6^ Moreover, composite specimens are more suited to direct analysis using cross-sectional scanning probe microscopy. For example, Yamamoto et al. recently demonstrated the use of amplitude-modulated AFM to directly detect soft regions surrounding silica nanoparticles embedded in an epoxy–amine resin (20 nm depth),^7^ while we applied subdiffraction limit infrared microscopy (AFM-IR) to demonstrate directly that an excess of epoxy is located very close (<50 nm depth) to buried iron oxide nanoparticles in epoxy resins cross-linked with aliphatic amine (triethylenetetramine, TETA).^6^

In the case of iron oxides, we previously coupled this analytical approach to computational modeling to determine the expected binding strength of the diglycidyl ether of bisphenol A (DGEBA) epoxy and TETA amine molecules. Generally, for physisorption via electrostatic interaction, the metal oxide surface structure was found to exert more influence than the nature of the adsorbing molecule. While amine adsorption was consistently weakly preferred over the epoxy or cross-link junctions, the strength of interaction varied more greatly with the iron oxide, from strong (goethite) to weak (hematite) to repulsive (magnetite) in molecular dynamics (MD) simulations.^6^ Surprisingly, however, the extent of undercuring was found to be independent of these predicted binding strengths and led us to propose that entropic segregation of the molecules drove the development of nanoscale chemical gradients, in keeping with the detailed atomistic simulations of interphase development at rigid surfaces reported by Yamamoto et al., which demonstrated that, in the absence of electrostatic binding, entropic segregation to a copper (charge neutral) interface prior to the cure yielded extensive (10 s nm) chemical gradients in epoxy–amine materials, which persist after cross-linking.^8,9^

Recently, we reported an extension of this approach to an alternative *m*-xylylene diamine (MXDA) cross-linker with goethite and hematite pigments to examine the influence of binding on network dynamics.^10^ For ambient cured resins, the results confirmed that the overall degree of undercuring in the network (signified by the retarded evolution of secondary hydroxyl groups) induced by the pigment was again insensitive to the binding energy (iron oxide type). Conversely, however, for MXDA, the level of residual primary amine remaining in resins could be assessed using infrared spectroscopy, and surprisingly, this was found to depend strongly on the predicted binding energies (i.e., the nature of the iron oxide pigment). In that case, when polymerization was performed at elevated temperature, no undercuring could be detected, indicating that the reaction kinetics and increased mobility of small molecules prevented extensive entropic segregation. However, even in the absence of a detectable effect on cure degree, the residual amine concentration continued to be depleted as a function of the added goethite content. Here, we examine this seeming contradictory finding in more detail, first seeking direct experimental validation of the computationally predicted binding energies and then applying FTIR and nanospectroscopy to DGEBA—*m*-xylylenediamine (MXDA) and computational modeling to cross-linked resin interphase regions formed in contact with goethite, hematite, and magnetite particles.

## Experimental Section

### MXDA Adsorption Experiments

0.2 g of iron oxide pigments was added to a 10 vol % solution of *m*-xylylenediamine (MXDA) hardener (99%, Sigma, used as received) in toluene (99%, Fisher), followed by mixing using an ultrasonic bath. The pigments were Bayferrox 318 M (>96% Fe_3_O_4_, Bayer), Bayferrox 140 M (>94% synthetic Fe_2_O_3_, Bayer), and Bayferrox 3920 (>99% synthetic Fe(O)OH, Bayer). We have previously reported scanning electron microscopy analysis of these pigment particles;^6^ further examples are given in Figure S1 in the Supporting Information. While goethite is of a relatively irregular acicular morphology with mean dimensions of 420 ± 190 nm × 80 ± 20 nm, we found that the globular hematite and magnetite particles are of comparable dimensions (210 ± 90 nm and 250 ± 110 nm diameter for magnetite and hematite, respectively). The pigments were separated from the MXDA solution by filtration, washed twice with toluene, and dried under nitrogen, before analysis using FTIR. ATR-FTIR spectra were obtained over the 500–4000 cm^–1^ range using an Alpha spectrometer (Bruker) equipped with a platinum diamond ATR accessory (Bruker) operating at 4 cm^–1^ resolution. Twenty-five co averages were added to each scan.

For UV–vis experiments, 10 mL 10 v/v % in toluene (99%, Fisher) solutions of MXDA were prepared, and 1, 2, 3, 4, and 5 vol % of pigment were added, followed by ultrasonic mixing for 30 min and filtration. Spectra were obtained in quartz cuvettes against a toluene reference by using a Lambda 25 UV Vis spectrometer (PerkinElmer).

### Composite Sample Preparation

**Scheme 1:**
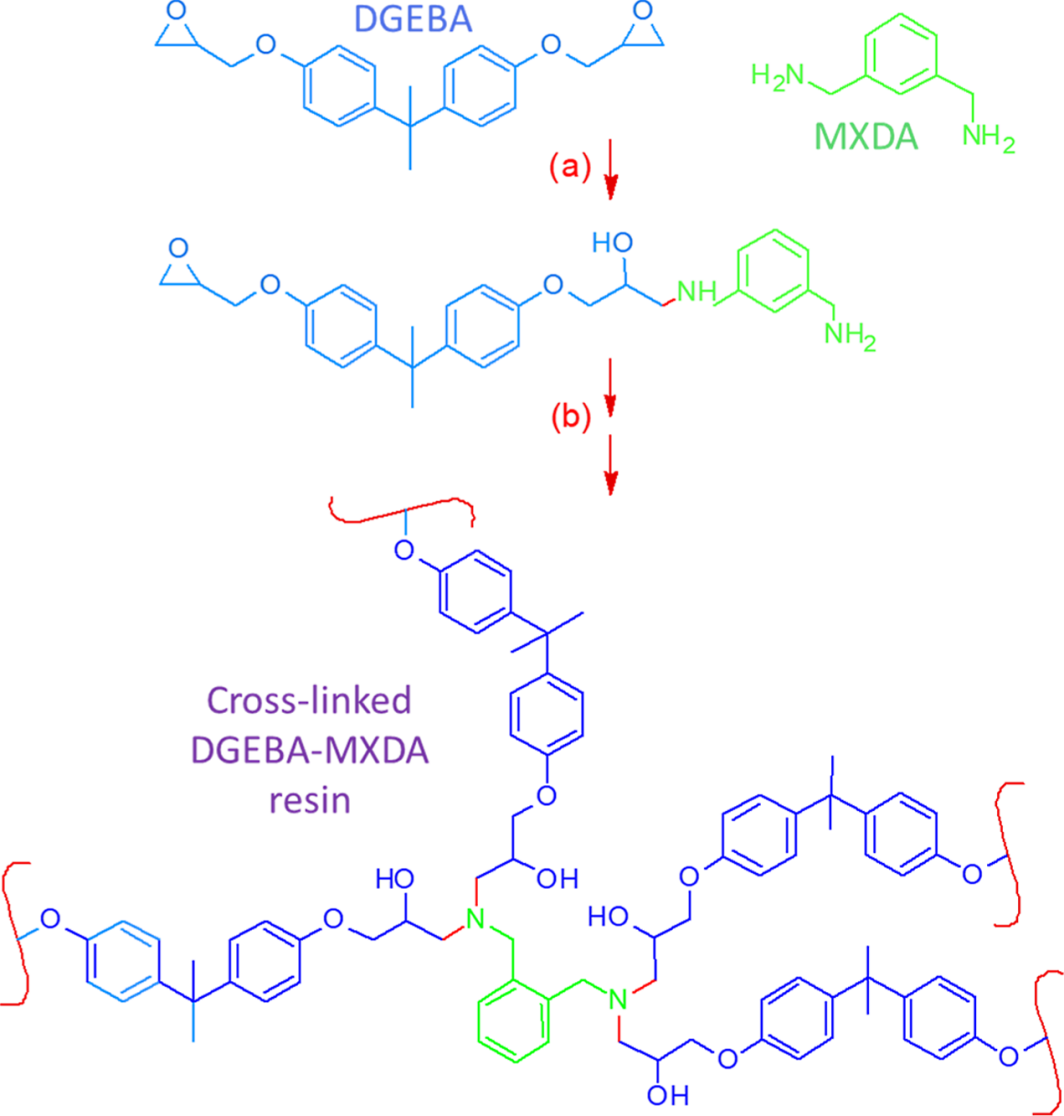
Cross-Linking Reaction of Diglycidyl Ether of Bisphenol-A (DGEBA) with *m*-Xylylene Diamine (MXDA)

Epoxy–amine resins consisted of stoichiometric mixtures of DGEBA epoxy (17.3 g of D.E.R. 332, Sigma-Aldrich, used as received, EEW 171–175 g/eq) and *m*-xylylenediamine (MXDA) hardener (3.4 g, 99%, Sigma, used as received, AHEW 34 g/eq), Scheme 1. Since minor stoichiometric shifts are probed in this work, to avoid any loss of material upon transfer from a second vessel, the epoxy, amine, and iron oxide powders were weighed directly into the dual-axis centrifuge pot. Since the volume of the amine/powder lies below the minimum mass tolerance for mixing in the appropriately sized pots, to ensure an even dispersion of particles, preweighed iron oxide pigment powders were dispersed in the epoxy component for 2.5 min at 2500 rpm (Speedmixer, Flacktek Inc.) before addition of the amine cross-linker and further mixing for 30 s at 1500 rpm to achieve final pigment volume concentrations between 0.5% and 3.5%. Note that although pigments are referred to as iron oxides throughout for simplicity, Fe(O)OH is an iron oxyhydroxide. Unless specified otherwise in the text, composites were allowed to cure under ambient conditions (22 °C), for 7 days, before postcure heating at 120 °C for 16 h. In order to expose the bulk structure, composite specimens were sequentially abraded using 600, 800, and 1200 grit silicon carbide grinding papers, with water as a lubricant. Samples were then thoroughly rinsed in deionized water, air-dried, and stored in a desiccator prior to analysis.

### Fourier Transform Infrared Spectroscopy

ATR (attenuated total reflection)-FTIR (Fourier transform infrared) spectroscopy of the composite epoxy–amine/iron oxide specimens was performed using an FTIR spectrometer (Nicolet 5700 spectrometer, Thermo Electron Corp.) equipped with a room-temperature DTGS (deuterated triglycine sulfate) detector operating at 4 cm^–1^ resolution across the 4000–500 cm^–1^ range. 64 coaverages were added to every spectrum.

### MD Simulations

Atomistic MD simulations of cross-linked films were performed using LAMMPS (Large-scale Atomic/Molecular Massively Parallel Simulator) software,^11^ with a constant temperature, volume, and number of particles (*NVT*). The system is integrated through time by the velocity-Verlet algorithm^12^ with a time step of 1 fs, and the temperature is controlled by the Nosé–Hoover Thermostat.^13^ Periodic boundary conditions are applied in the plane of the substrate surfaces (the *x* and *y* directions). Long-range electrostatic corrections are calculated using the particle–particle–particle–mesh (PPPM) solver,^14^ with slab corrections for systems with periodicity in only two dimensions.^15^

Crystallographic data was used to construct iron oxide slabs.^16−18^ Surfaces were oriented normal to the *z*-axis, with the surface face hematite (0 0 0 1) and goethite (1 0 0) chosen as the most thermodynamically stable.^19,20^ Magnetite (0 0 1) was chosen as the dominant growth face.^21,22^ The ClayFF force field^23^ with Kerisit modifications to Fe atoms^24^ is used to model surface atom interactions. To accelerate the simulations, surface atoms are fixed in place throughout. For monomers (DGEBA and MXDA), the All-Atom Optimized Potentials for Liquid Simulations (OPLS-AA) force field was used.^25^ Monomer structure and force field parameters were generated by the online tool LigParGen,^26^ with atomic point charges calculated by the 1.14*CM1A algorithm, see Table S1 and Figure S1, Supporting Information.^27^ An initial randomly packed monomer system is created by Packmol^28^ and Moltemplate^29^ with a simulation box of approximately 80 Å in the plane of the surface (*x* and *y*), to match the surface slab dimensions, and 100 Å normal to the surface in the *z* dimension. To maintain a stoichiometric ratio, 400 DGEBA molecules and 200 MXDA molecules were packed into the system. Three repeats were performed for each surface with unique randomly packed starting structures over which results are averaged.

**Scheme 2:**
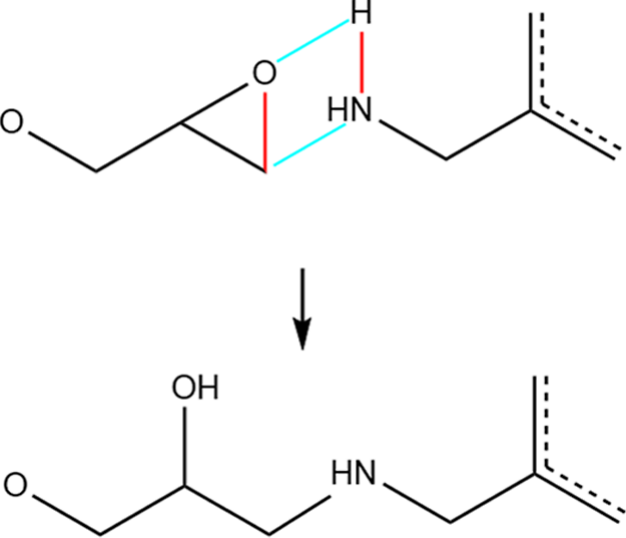
Crosslinking Reaction of DGEBA with Primary MXDA Amine Groups as Implemented in Simulations During the reaction, blue bonds are created and red bonds are destroyed. The equivalent reaction is performed on secondary MXDA amine groups, with the amine hydrogen atom not involved in reaction replaced with a polymer chain.

Prior to the introduction of the surface slab or cross-linking reactions, the monomers were mixed and equilibrated according to the procedure of Demir and Walsh,^30^ whose details are provided in Table S2 in the Supporting Information. Following mixing, the surface slabs are introduced directly underneath the equilibrated monomer mixture and allowed to equilibrate for 1.5 ns. Cross-linking reactions are then performed using the REACTER code available in LAMMPS.^31,32^ The reaction scheme is shown in Scheme 2. Every 1000 fs, a reaction is attempted on all pairs of reactive atoms (DGEBA terminal epoxy carbon and MXDA nitrogen) within *r*_cut_ = 3.5 Å. When a reaction takes place, bonds are instantaneously destroyed and created, and atom point charges are reassigned based on the product structure. The probability of a reaction taking place between atoms within *r*_cut_ is determined by the local temperature *T* and activation energy *E*_a_ using the Arrhenius equation , where *R* is the gas constant and *A* is the arbitrary constant. The activation energies used are 13.35 and 12.95 kcal/mol for the primary and secondary reactions, respectively, according to experimental measurements.^33^ To achieve a rate of reaction on simulation time scales without excessively short reaction times, *A* = 5.5 × 10^6^ was used. Cross-linking was performed at 323 K for 7 ns, after which the reaction rate decreased significantly around 60% conversion, necessitating a high-temperature postcure of 500 K for a further 8 ns to reach conversions around 80%. Finally, the cross-linked structure was annealed at 600 K for 3 ns, cooled to 300 K over 15 ns, and simulated for 1.5 ns at a constant 300 K. Structural analysis was performed over the final 1 ns.

The Python package MDAnalysis^34^ was used to calculate linear concentrations, which were plotted by MatPlotLib.^35^ Rendered images of simulated systems were produced by VMD.^36^

### AFM-IR Analysis

For nanoanalysis, polymer cross sections of 100 nm nominal thickness were prepared using an ultramicrotome (Leica EM UC6) with a diamond knife. Cross sections were collected on transmission electron microscopy (TEM) grids (Agar Scientific), which were then floated onto a droplet of deionized water placed on a ZnS substrate (Anasys Instruments). Upon evaporation of the droplet, TEM grids were removed, specimen sections remained on the ZnS surface, and these were dried for >16 h in a desiccator prior to examination.

During AFM-IR analysis, the microtamed sections were illuminated by a pulsed tunable QCL laser. Subdiffraction limit resolution is achieved by monitoring the deflection of an AFM probe in contact with the surface. This results from rapid transient thermal expansion of the material in contact with the probe tip in response to infrared absorbance.^37^ The recorded AFM-IR signal is the amplitude of the induced AFM probe oscillation, obtained after a fast Fourier transform. Stepping the incident radiation through the infrared fingerprint region and recording the amplitude in this way has previously been shown to generate spectra closely matched to those measured using conventional macroscopic FTIR.^38^ For imaging, simultaneous contact-mode topographical measurement and infrared mapping can be performed by holding the incident laser at a given wavelength during scanning.^39−42^ In the present study, AFM-IR images were collected on a NanoIR3 system (Bruker) in resonance-enhanced contact mode at a scan rate of 0.5 Hz using a gold-coated silicon nitride probe (0.07–0.4 N/m spring constant, 13 ± 4 kHz resonant frequency, Bruker). The pulse rate, maintained at a repetition rate of approximately 180 kHz via a PLL feedback loop (matched to the resonant frequency of the cantilever) and the amplitude of infrared-induced oscillation, was recorded at a given wavelength using 256 points per 128 scan lines. Sets of images were collected in at least three different regions to ensure reproducibility.

### Dynamic Scanning Calorimetry

For dynamic scanning calorimetry (DSC) measurements, 6–10 mg specimens were placed in closed aluminum pans. DSC thermograms were obtained using a heat–cool–heat cycle over a temperature range of 0 to 200 °C under nitrogen using a heating/cooling rate of 10 °C min^–1^/5 °C min^–1^ (Q100 DSC, TA Instruments). *T*_g_ measurements were obtained from the second heat cycle in all cases.

## Results and Discussion

### Pigment–Amine Binding

**Figure 1:**
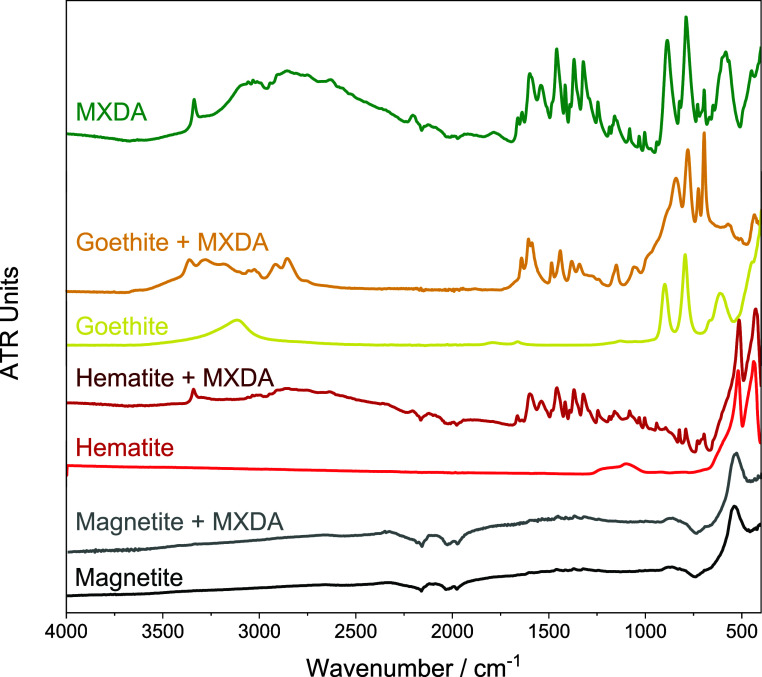
FTIR-ATR spectra obtained for MXDA as received (top) and pairs of spectra corresponding to goethite, hematite, and magnetite pigment powders before (bottom) and after (above) exposure to MXDA solution, washing, and drying.

Previously reported MD simulations predict that the preferential immobilization of amines on iron oxides follow the order goethite > hematite > magnetite (binding to hematite was found to be more energetically favored due to the unfavorable molecular conformations required for bidentate binding to goethite sites. However, adsorbed molecules were significantly less mobile on goethite).^6,10^ Here, experimental confirmation of this relationship was sought by measuring the degree of MXDA adsorption onto the pigment powders from the solution. First, for infrared analysis, 0.2 g of pigment was added to an excess of amine in solution (100 mL of 10% (v/v) MXDA in toluene). This was followed by 30 min mixing in an ultrasonic bath, filtration, extensive washing in toluene, and drying under vacuum. Comparison of the FTIR-ATR spectra obtained for the virgin pigment and the dried, exposed powders clearly demonstrates significant retention of MXDA on goethite and hematite, while almost no signal related to the amine was detected on magnetite particles, Figure 1. It is notable that the absence of amine retained by magnetite is in keeping with the expected repulsive interaction between adsorbing molecular amines and the magnetite surface, as predicted by MD simulations, whereas electrostatic binding between MXDA and the goethite and hematite surfaces is expected.

**Figure 2:**
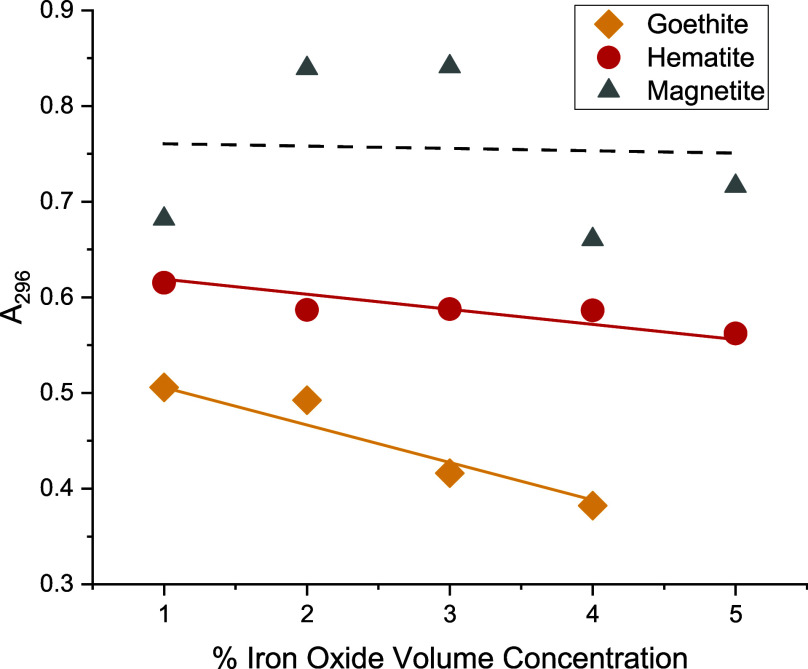
UV–vis absorbance maxima of 10% MXDA solutions in toluene at 296 nm following the addition and removal of pigment powder, as a function of the added pigment volume concentration for goethite, hematite, and magnetite powders.

Second, UV–vis analysis of amine solutions (10 v/v % in toluene) was performed against a toluene reference following the addition of 1–5 vol % of pigment to solutions, ultrasonic mixing for 30 min, and filtration. Note that since the UV spectra for solutions exposed to magnetite were largely saturated, the results are extremely noisy. Nonetheless, comparison of the absorbance maximum at 296 nm provides confirmation that amine depletion from solutions exposed to pigment powders followed the order of goethite > hematite > magnetite, Figure 2.

### Composite Specimen Infrared Spectroscopy

**Figure 3:**
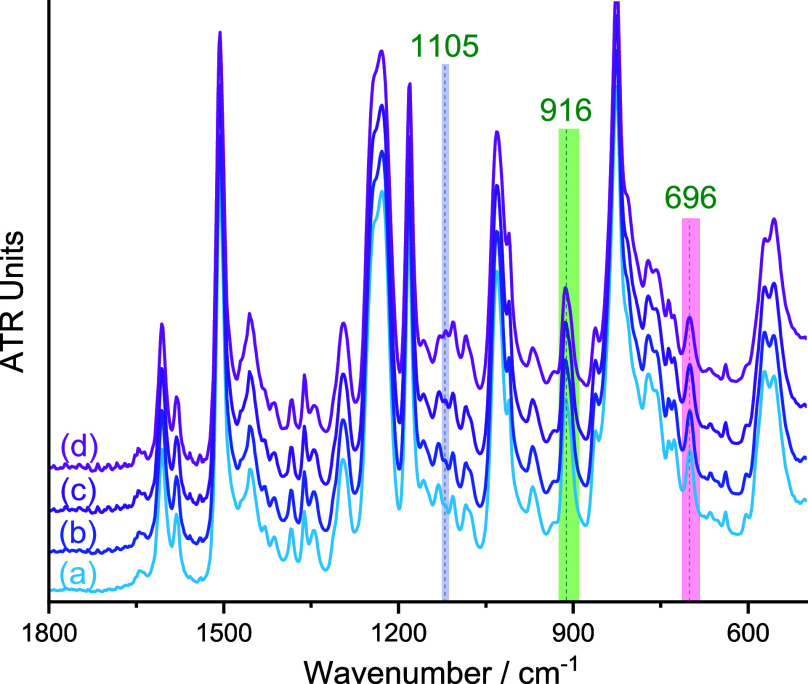
ATR-FTIR spectra of stoichiometric DGEBA–MXDA mixture (in the absence of iron oxide pigments) (a) 32; (b) 64; (c) 180; and (d) 301 min after mixing.

To monitor DGEBA–MXDA network development in composite samples, infrared bands previously identified as characteristic of the epoxy–amine cure were used, namely, the asymmetric oxirane ring deformation at 916 cm^–1^, corresponding to epoxy groups,^43,44^ the C–N–H wag of the primary amine at 696 cm^–1^^10,45^ (also) consumed during the cure, and the out-of-phase C–C–O stretch for secondary alkyl hydroxy groups at 1105 cm^–1^, which are generated by the cross-linking reaction.^46^ Integration of these infrared bands, highlighted in Figure 3, was followed by normalization to the area of the aromatic band at 1504 cm^–1^ and used to measure and compare the effect of iron oxides on the overall cure progression of the DGEBA–MXDA matrix.

**Figure 4:**
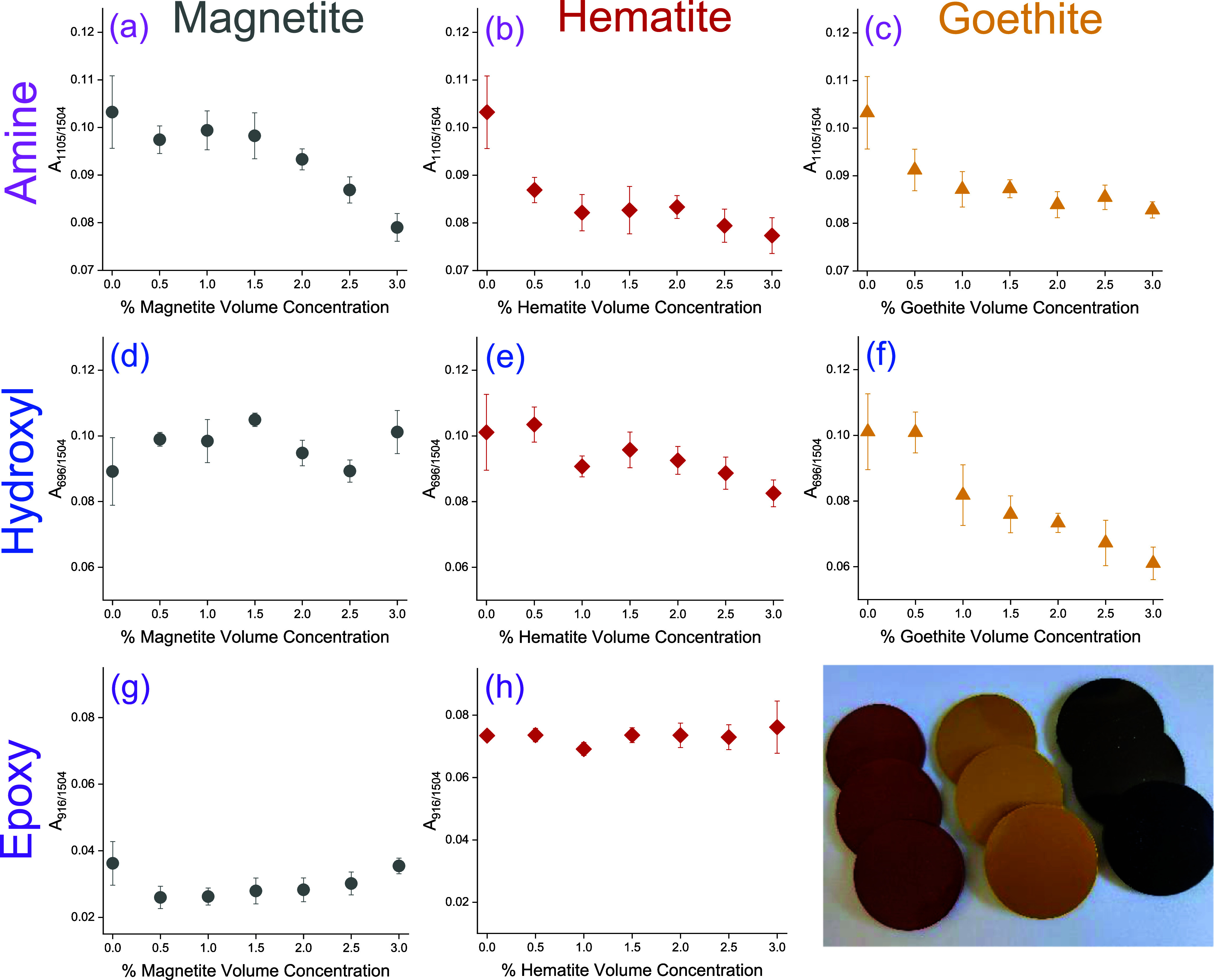
Normalized area of the FTIR-ATR bands for composite specimens cured for 7 days under ambient conditions, followed by 16 h postcure heating at 120 °C, corresponding to the secondary hydroxyl band at 1105 cm^–1^ (top row) as a function of pigment volume concentrations for (a) black magnetite; (b) red hematite; and (c) yellow goethite pigments, the residual amine band at 696 cm^–1^ (middle row) as a function of pigment volume concentrations for (d) black magnetite; (e) red hematite; and (f) yellow goethite pigments, and the residual epoxy band at 916 cm^–1^ (bottom row) as a function of pigment volume concentrations for (g) black magnetite and (h) red hematite pigments. Points correspond to the mean of five individual measurements, and error bars correspond to one standard deviation. Bottom right: digital photograph of composite specimens.

In all cases, the concentration of secondary hydroxyl groups produced by cross-linking is diminished as a function of the added iron oxide volumes, indicating that network development is retarded by the presence of all pigments during the ambient cure, Figure 4a–c. This is consistent with our previous works demonstrating that epoxy sequestration in the interphase is largely insensitive to binding strength and likely occurs as a consequence of entropic segregation.^6^ Moreover, we have previously shown that this effect is eliminated by curing at 120 °C for DGEBA–MXDA systems.^10^ Here, extended postcure heating regimes were employed (16 h at 120 °C compared to 2 h at 120 °C in our previously reported DGEBA–MXDA study) to examine whether the underdeveloped regions dissipate due to thermally induced diffusion of small molecules. This temperature was selected since it falls below the temperature where etherification/thermal degradation is expected and allows direct comparison to the previous work. From these results, however, it appears that once a partially cured network structure is established under ambient conditions, it persists on heating due to limited mobility of the partially cured material in the interphase region.

In contrast, the measured residual amine (in an unbound state, note that binding restricts the C–N–H wag vibration) was found to match well with the immobilization on iron oxides predicted by MD simulation and adsorption measurements, i.e., there is no discernible correlation to added magnetite volumes (Figure 4d), an inverse relation to the added hematite volumes (Figure 4e), and a stronger inverse dependence to added goethite volumes (Figure 4f). This is in keeping with previous results, where we demonstrated that these correlations persist even when no underdevelopment of the network is observed following high-temperature curing.

As a consequence of the prolonged postcure heating time, in the case of hematite and magnetite composites, no clear trends could be discerned for residual epoxy groups as a function of added pigment volume, Figure 4g,h. Note that for goethite, this band is, however, overlapped by a pigment absorbance; thus, integration is not possible; see Figure 1. The absence of a trend may be due to noise in the data since the absorption band is very weak. Nonetheless, it is apparent that more residual epoxy is present in the hematite-filled specimens than for magnetite. In keeping with the correlation to residual amine content, this indicates that under ambient conditions, retarded network development in the interphase is not purely determined by entropic segregation of the molecular constituents, but that interfacial sequestration of the amine plays a pivotal role.^6^ Further insights into the development and structure of the interphase regions were thus sought using MD simulation.

### MD Simulation

**Figure 5:**
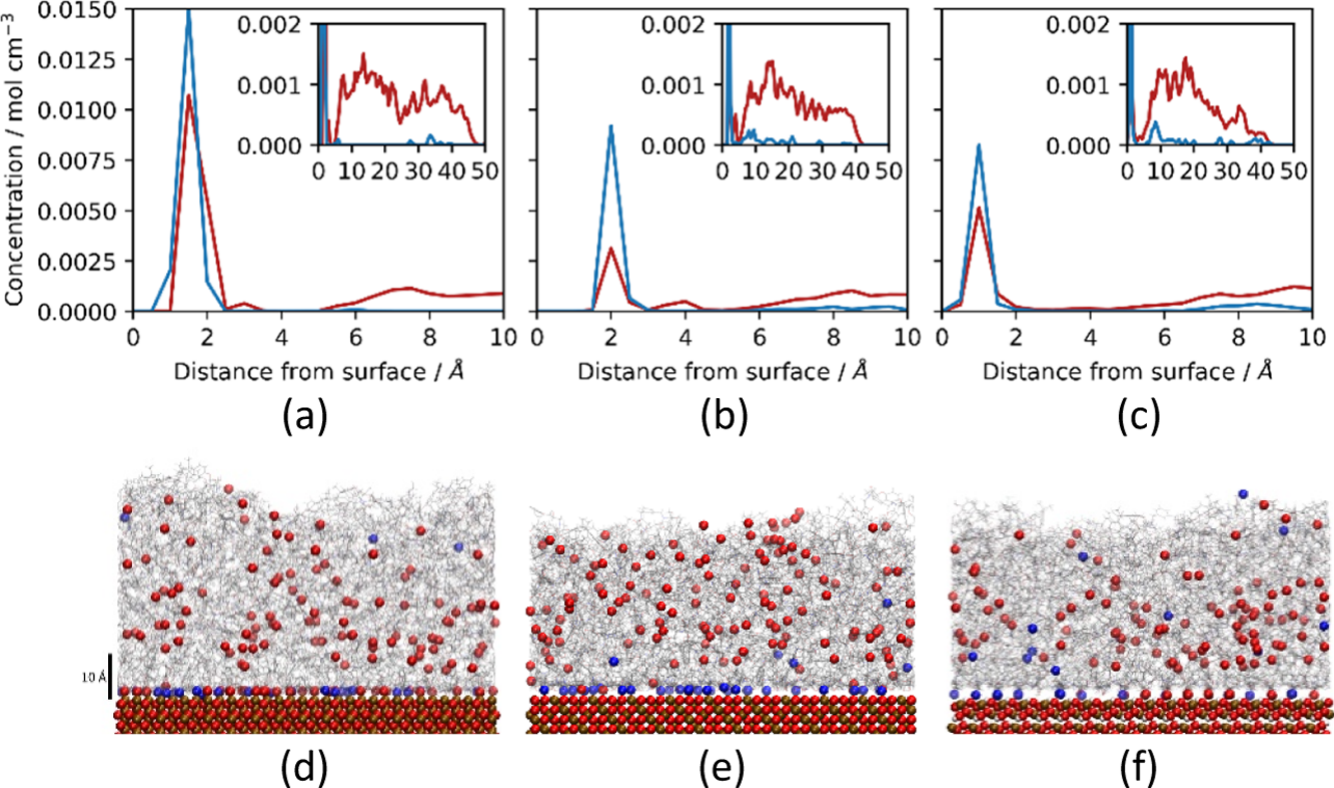
(a–c) Linear concentration plots of unreacted DGEBA epoxy oxygen atoms (red) and unreacted MXDA primary amine nitrogen atoms (blue) as a function of distance from the topmost (a) hematite, (b) magnetite, and (c) goethite surface atoms. The main plots focus on the peak in concentration at the surface contact layer, while insets show the overall profile throughout the film. Additional plots are available in the SI. (d–f) Corresponding renders of DGEBA/MXDA systems cross-linked at (d) hematite, (e) magnetite, and (f) goethite after relaxation to 300 K. The entire film structure is shown in gray, while unreacted epoxy oxygen atoms are indicated with red spheres, and unreacted primary amine nitrogen atoms are indicated with blue spheres. Surface iron, oxygen, and hydrogen atoms are shown as ochre, red, and white spheres, respectively. A scale bar is included beside (d) to show a 10 Å length in (d–f).

Insight into the development and persistence of interphase regions was obtained using all-atom MD simulations to model the polymer structure at the nanometer scale. To approximate network formation under ambient conditions (prior to postcure heating), cross-linked DGEBA/MXDA networks were formed up to 80% conversion in contact with hematite, magnetite, and goethite surfaces since it is well established that epoxy–amine curing does not progress to completion under ambient conditions. After the partially cured networks were relaxed, concentration distributions were calculated for unreacted DGEBA epoxy oxygen and primary MXDA nitrogen atoms to highlight regions of unreacted epoxy or amine functionalities. These distributions are shown in Figure 5.

**Figure 6:**
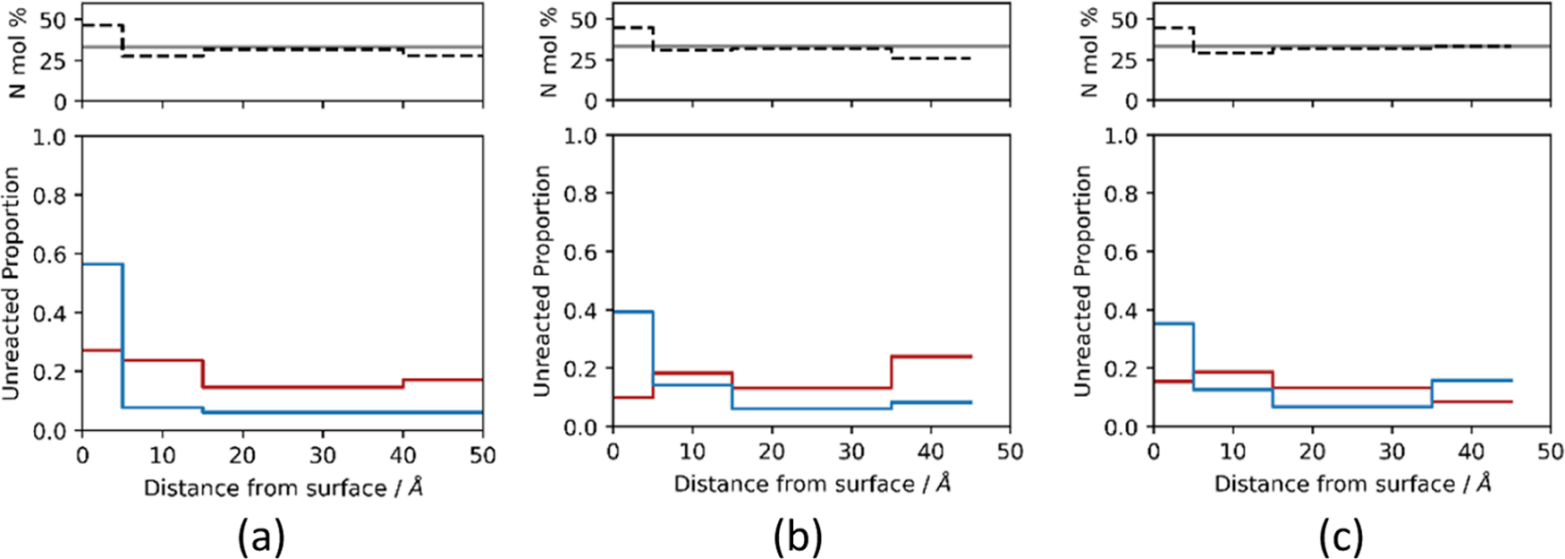
Fractions of unreacted (blue) amine and (red) epoxy functionalities calculated in bins at 0, 5, 15, 35, and 45 Å distance from (a) hematite, (b) magnetite, and (c) goethite surface atoms (the last two bin edges are shifted to 40 and 50 Å for hematite owing to slightly different surface dimensions). Secondary amines are treated as “half-reacted” functionalities. Above each plot, the percentage MXDA composition is shown for each bin as a dotted line. A solid line indicates the value at the stoichiometric ratio.

Significant quantities of both unreacted primary amines and epoxies in the surface contact layer are evidenced by the strong initial peaks due to low mobility of adsorbed species (the peaks for amine are higher than the peaks of epoxy, despite the overall composition of 2 epoxy molecules per each amine molecule). The concentration profiles of the individual primary, secondary, and tertiary amines (Supporting Information, Figures S3–S4) show that fully reacted tertiary amines are found far away from the surface, whereas primary and secondary amines accumulate at the interface, with secondary amines being further away from the surface for magnetite and goethite. There is also a clear peak of unreacted epoxy near the surface (Supporting Information and Figure S5). Figure 6 shows the fraction of unreacted amine and epoxy as a function of the distance from the surface. The fraction of unreacted amines near the surface is between 0.35 and 0.6, whereas the fraction of unreacted epoxy to total oxygen is between 0.1 and 0.3. This shows that most of the amines near the surface have not fully reacted, whereas most of the epoxy groups have reacted. In the interphase further from the surface, there is a depletion of primary amine due to the preferential adsorption of MXDA to the surface observed in previous simulations.^47^ This results in a region of excess uncured epoxy functionalities, as observed in the insets of Figure 5a–c. Renders of the systems are shown in Figure 5d–f, in which unreacted epoxy and primary amine functionalities are indicated to highlight these distinct regions. Note that, importantly, the spatial separation of the regions of primary amine and epoxy excess means that at high cures where the polymer has gelled, even the increased mobility of these species at high temperatures during postcure heating is not expected to enable these groups to meet and react. This is in contrast with the previous work, where we have shown that the mobility of precursors during the early stage of the cure (i.e., performing the entire cure at 120 °C) eliminates detectable under-reaction.^10^

### AFM-IR Analysis

**Figure 7:**
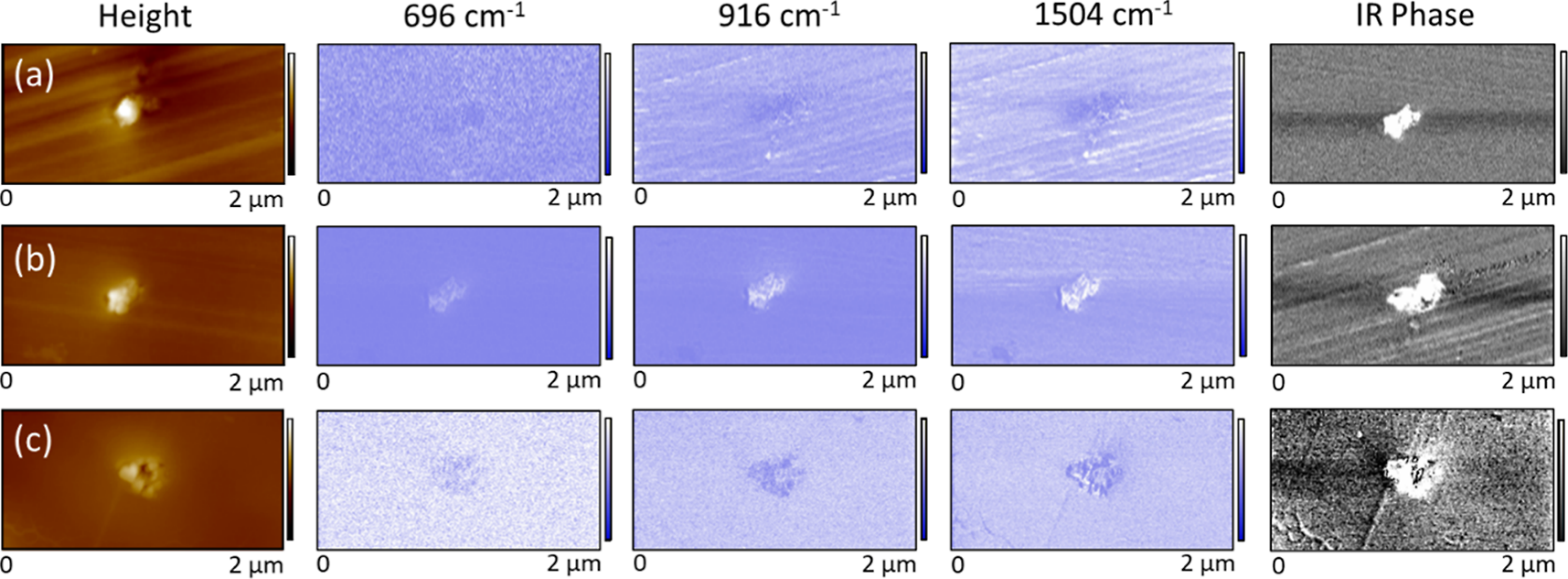
Two μm × 1 μm AFM-IR contact mode height images (*z* scale 100 nm) alongside the corresponding IR-induced amplitude maps obtained using incident infrared illumination at 696, 916, and 1504 cm^–1^ (*z* scale 0.5 mV) and IR phase maps obtained at 1504 cm^–1^ (*z* scale 90°). Images show pigment agglomerations in 100 nm-thick cross-sectional samples of DGEBA–MXDA epoxy amine resins containing 1% volume concentrations of (a) hematite; (b) goethite; and (c) magnetite.

The location of residual epoxy and amine concentrations was confirmed for postcured resins using AFM-IR analysis operating in high-resolution resonance-enhanced mode. 100 nm-thick cross sections were prepared using ultramicrotomy, and the region surrounding pigment particles was mapped at 696, 916, and 1504 cm^–1^ corresponding to residual amine groups, unreacted epoxy groups, and aromatic groups, respectively, see Scheme S2 in the Supporting Information for the AFM-IR setup. For hematite and magnetite, the IR-induced amplitude signal was found to be lower directly over the mineral pigment; see Figure 7a,c. This is expected for pigmented systems since the volume of the IR absorbing polymer underneath the AFM probe tip is reduced (note that the binder beneath/around the particle is expected to contribute to the signal).^6,48,49^ In the case of goethite, the IR signal is consistently raised directly over the pigment particle, as shown in Figure 7b. Since the contrast generated is strongest where the incident wavenumber coincides with a goethite absorbance band (at 916 cm^–1^), it is likely that this enhanced signal is due to thermal expansion contributions from the IR absorbance of the pigment particle itself. In keeping with this, the IR phase maps (where the phase lag of infrared-induced vibration vs the incident pulse is indicative of mechanical properties^50^) indicate that in all cases, the pigment is harder than the surrounding resin, and no soft partially cured region is detected.

**Figure 8:**
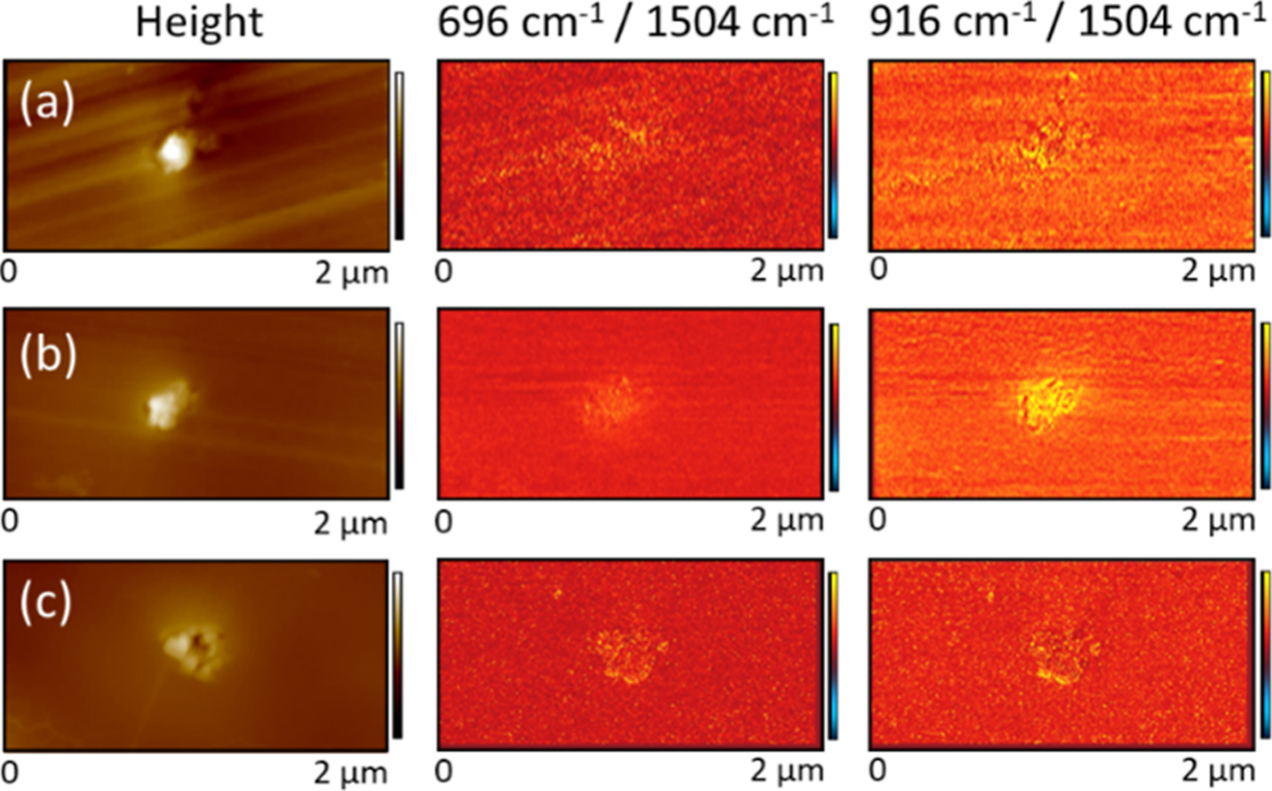
Two μm × 1 μm AFM-IR contact mode height images (z scale 100 nm) alongside the corresponding IR ratio image generated from IR amplitude maps at 696 cm^–1^/1504 cm^–1^ and 916 cm^–1^/1504 cm^–1^ (*z* scale 5). Images show pigment agglomerations in 100 nm thick cross-sectional samples of DGEBA–MXDA epoxy amine resins containing 1% volume concentrations of (a) hematite; (b) goethite; and (c) magnetite.

To account for these differences in sampling volume and probe response, ratio images were produced by dividing the raw IR amplitude data gathered at 696 and 916 cm^–1^ with that generated at 1504 cm^–1^, cross-correlated using the respective height scans. This process is akin to spectral normalization to the aromatic band and allows visualization of the distribution of amine, epoxy, and hydroxyl groups.^51^ While the effect is small, ratio images generated in this manner over at least three pigment particles per sample consistently showed an increase in signal for both residual epoxy and, surprisingly, contrary to the bulk FTIR analysis and MD simulations, increased residual (unbound) amine directly over the pigment particles too, Figure 8. However, note that the absence of a detectable amine-depleted region in AFM-IR spectra may lie in its low concentration throughout the resin. Furthermore, since the lateral resolution for AFM-IR imaging has not been defined for these specimens, and moreover, smearing/spreading/loss of the partially reacted material during sectioning and transfer cannot be ruled out, the extent and depth of the interphase formed in each case can neither be determined nor be compared qualitatively using this approach.

### Chemical Interphase Development

**Figure 9:**
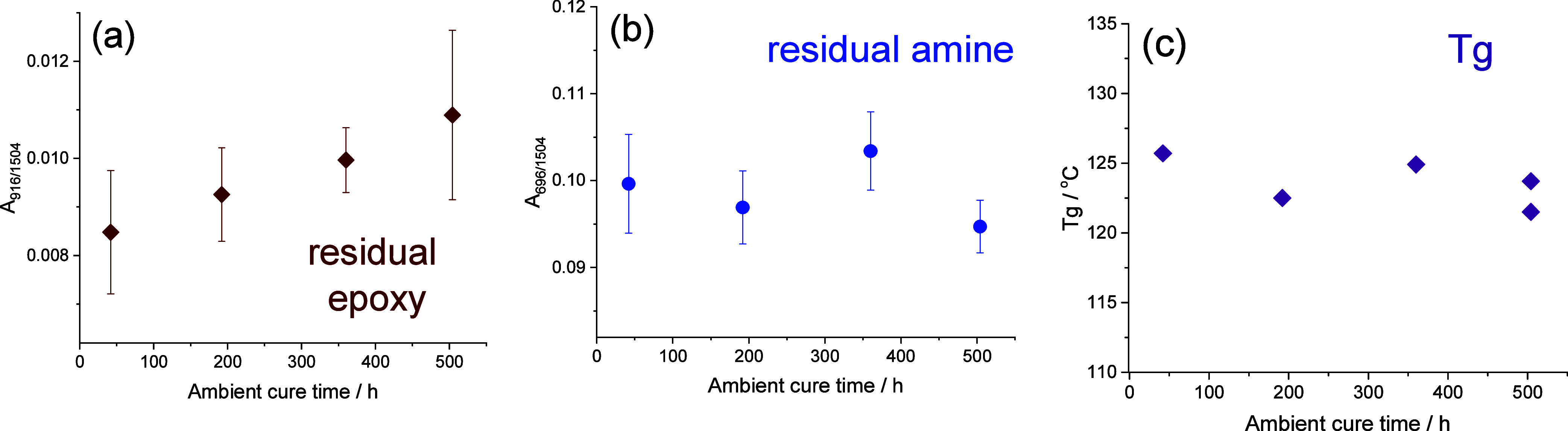
(a) Normalized areas of the residual epoxy infrared band at 916 cm^–1^; (b) normalized area of the residual primary amine infrared band at 696 cm^–1^, and (c) measured *T*_g_ for DGEBA–MXDA specimens containing 3% v/v hematite powder as a function of ambient cure time prior to 2 h postcure heating at 120 °C. For (a,b), each point corresponds to the mean of 10 individual measurements and error bars correspond to one standard deviation.

We have previously reported that high-temperature curing eliminates the excess epoxy and variations in cure degree detectable by FTIR-ATR in the presence of iron oxides, indicating that at high temperatures, diffusion of excess epoxy segregated on mixing occurs into the bulk material, enabling its reaction to below detectable levels. Conversely, the analysis here indicates that unreacted groups that segregate during the slower, ambient temperature cure are trapped by segregation prior to complete reaction and thus remain at detectable levels. Taken together, these results indicate that the interphase development is a kinetic effect, where the effect of preferential amine binding on local properties is dependent on the ability of excess epoxy groups to diffuse away from the interphase region during the cure. Thus, the impact of the ambient cure time was assessed. Residual epoxy and amine contents were assessed as a function of ambient cure time for MXDA–DGEBA resins containing 3% v/v hematite particles using ATR-FTIR. For this experiment, specimens were allowed to cure under ambient conditions for 17 h–21 days, prior to a postcure heating for 2 h at 120 °C under nitrogen (the shorter postcure heating allowing the detection of residual epoxy groups). Integration of the infrared bands revealed that the concentration of residual epoxy groups remaining in the resins clearly increases as a function of ambient cure time, while no trend could be discerned for the amine, Figure 9a,b. Furthermore, *T*_g_ values of the bulk resin were unaffected by ambient cure time, and no indication of residual cure was detected in DSC thermograms (Figure 9c). This, when taken alongside the results presented above, strongly indicates that the excess epoxy measured does not reflect decreased cross-linking of the bulk matrix and is primarily located in the interphase regions.

The continued increase in excess epoxy functionality as a function of ambient cure time (i.e., postgelation) can be explained by the slow entropic segregation of the uncured/partially cured material toward iron oxide interfaces. While entropic segregation can be understood as occurring due to the difference in packing density, size, etc., of the precursor molecules prior to the cure (i.e., as a consequence of the precursor molecular structure), the partitioning of short-chain polymers to surfaces, to lower the overall entropic penalty of a rigid interface in polydisperse polymer systems, has long been established and is more pronounced as the molecular weight difference between chains increases.^52,53^ In accordance with this mechanism, it can be envisioned that the migration of the partially cured or uncured material to the interface regions continues as a kinetically controlled process as the molecular weight of the network polymer grows. This may be expected, since post gelation, the difference in flexibility, mobility, and packing ability between the macromolecular network and unreacted/partially reacted species will vastly increase. This mechanism may also explain the increase in residual epoxy as a function of ambient cure time, whereas the lower overall concertation of primary amine groups, and their preferential binding to the surface, negates any observable correlation.^54^

**Scheme 3:**
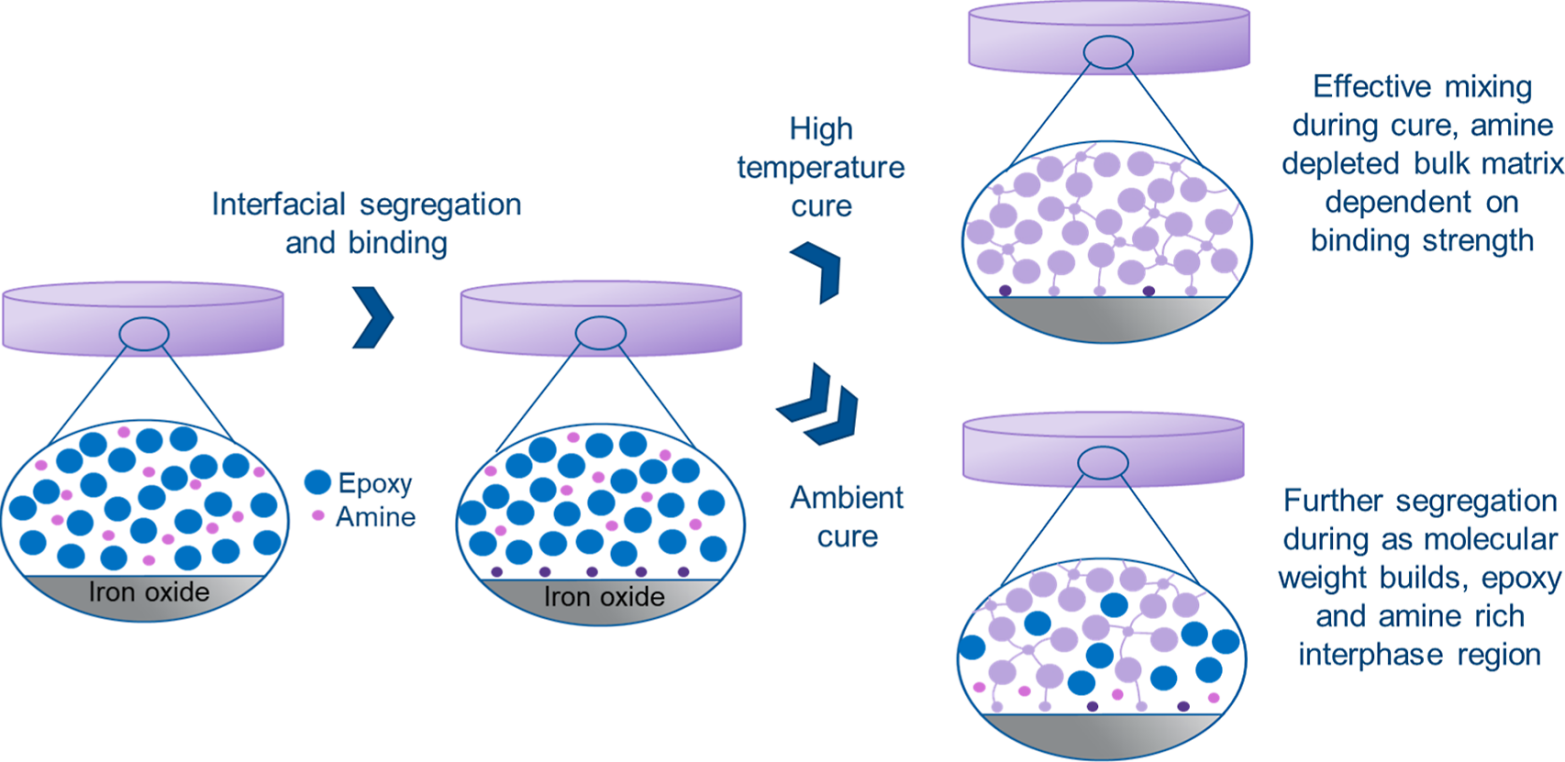
Development of Chemical Gradients in the Interphase in DGEBA–MXDA Resins Formed in Contact with Iron Oxide Surfaces: At High Cure Temperatures, Rapid Amine Binding Is Followed by Mixing, Leading to an Amine-Depleted Bulk Matrix Under ambient conditions, further entropic segregation of unreacted material occurs as molecular weight builds, leading to the accumulation of amine and epoxy groups in the interphase, which can be detected using AFM-IR.

In summary, Scheme 3 illustrates the proposed mechanism for the development of chemical gradients in the interphase of epoxy–amine materials. First, as indicated by absorption experiments and simulation, upon mixing, amines bind to the pigment surfaces when this is energetically favored. At high temperatures, effective mixing prior to the cure then yields an amine-depleted unreacted bulk matrix, in accordance with the relative binding strength. At the same time, entropic segregation of the molecular constituents will generate a chemical gradient of amine-rich and amine-depleted (epoxy-rich) regions close to the interface (nm scale), and under ambient conditions, this accumulates as the molecular weight builds, leading to an extensive partially reacted region which is detectable by scanning probe microscopy (10 s nm). This process, and thus the extent of chemical gradient developed in the interphase regions (i.e., beyond the contact layer), is considered to be primarily dependent on the molecular mobility vs cure kinetics.

## Conclusions

We have shown that previously reported MD simulations based on electrostatic interaction correctly predict the relative binding strength between diamine molecules and iron oxide surfaces. Moreover, for composites based on epoxy–amine binders, this relative binding strength is found to dictate amine depletion in the bulk matrix, as a result of effective mixing during the cure. This has implications for the design of coatings and composites based on epoxy–amine chemistry, where the macromolecular structure of the network polymer is often key to the overall performance. The strength of amine binding was, however, found to have little influence on the development of chemical gradients close to embedded pigment particles when compared to the cure temperature. Using detailed FTIR and AFM-IR analyses, we find that interphase development is instead dominated by segregation of molecular constituents and oligomers prior to and during the ambient temperature cure, which are shown to be spatially segregated within the interphase region in atomistic MD simulations. Even in the case of “fully cured” resins subjected to substantial periods of postcure heating above *T*_g_, particles remained embedded in a locally underdeveloped matrix, rich in reactive functional groups.

## Supporting Information Available

Additional experimental details, materials, and methods, including a schematic of the AFM-IR setup, electron microscopy images of the iron oxide particles, and MD parameters and simulation conditions used (PDF)
