# Supplementary material for: Probing the Nanostructure and Reactivity of Epoxy–Amine Interphases
Source: ACS Appl Mater Interfaces. 2024 Dec 9;16(50):70097–107. doi: 10.1021/acsami.4c17387 (PMC11660148; doi:10.1021/acsami.4c17387)
Supplement: Supplementary file 1 — am4c17387_si_001.pdf [file am4c17387_si_001.pdf]

Supporting Information

Probing the Nanostructure and Reactivity of Epoxy-Amine Interphases

Suzanne Morsch[1*], Yanwen Liu[1], Kieran Harris[2], Flor R. Siperstein[2], Claudio Di Lullo[3], Peter Visser[4] and Stuart Lyon[1]

1. Corrosion@Manchester, Department of Materials, The University of Manchester, Nancy Rothwell Building, Oxford Road, Manchester, M13 9PL, UK
2. Department of Chemical Engineering, The University of Manchester, Nancy Rothwell Building, Oxford Road, Manchester, M13 9PL, UK
3. AkzoNobel Powder Coatings, Stoneygate Lane, Felling, Gateshead, Tyne & Wear, NE10 0JY, UK
4. AkzoNobel, Rijksstraatweg 31, 2171 AJ, Sassenheim, The Netherlands

Contents

Scanning Electron Microscopy

Figure S1: Scanning electron microscopy images of iron oxide particles. S2

Molecular Dynamics Parameters & Molecular Charges

Table S1: Non-bonded parameters used for corresponding atom types during molecular dynamics simulations S3

Figure S2: Skeletal structures of DGEBA, MXDA, secondary amine fragment and tertiary amine fragment. S4

Simulation Conditions

Table S2: Simulation procedure. S5

Linear Concentration Plots

Figure S3: Linear density plots of the entire crosslinked film, DGEBA molecules and MXDA molecules. S5

Figure S5: Linear concentration plots of primary, secondary and tertiary amine nitrogen atoms. S6

Figure S5: Linear concentration plots of unreacted epoxy oxygen and reacted alcohol/epoxy product oxygen atoms S6

Scheme S1: The AFM-IR set-up S6

* Corresponding author email address: suzanne.morsch@manchester.ac.uk

Scanning Electron Microscopy

The microstructure and morphology of the iron oxide powders (synthetic hematite, Fe_2O_3, magnetite, Fe_3O_4 and goethite, Fe(O)OH) were characterized using scanning electron microscopy (SEM). A Zeiss Sigma field emission gun (FEG) SEM at 0.5 kV electron accelerating voltage was employed for this work. Iron oxide powders were placed on SEM specimen stubs and pressed against another SEM stub to form packed uniform layers of the iron oxide powders. They were gently blown at to remove any loose powder particles prior to placing them into the SEM chamber.

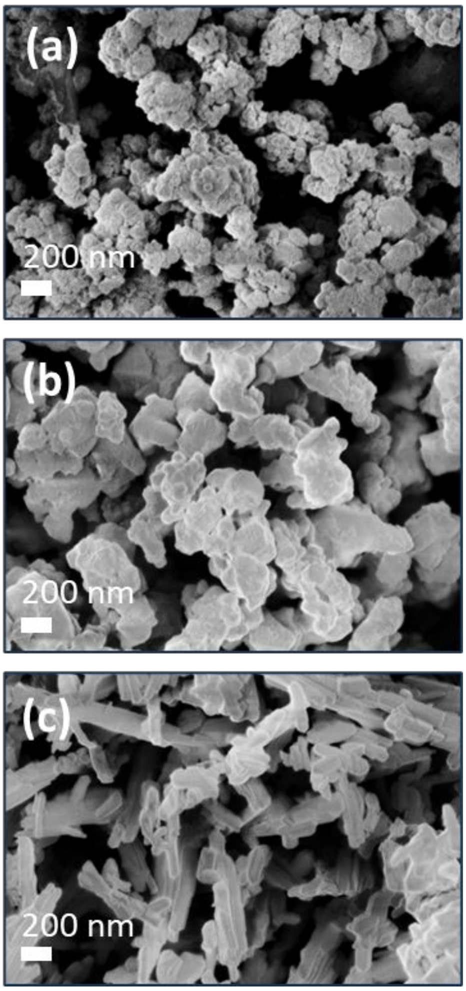

Figure S1. Scanning electron microscopy images of (a) magnetite (b) hematite and (c) goethite powders used in composite specimens.

Molecular Dynamics Parameters & Atom Charges

Table S1. Non-bonded parameters used for corresponding atom types during molecular dynamics simulations, as described by the OPLS-AA force field (organic molecules) and ClayFF with Kerisit modifications (iron oxides). All cross-interactions use standard Lorentz-Berthelot mixing rules.

Atom Type	Moltemplate ID	ε / kcal/mol	σ / Å	Charge
Alkyl C	80	0.066	3.50	-
Alkyl H	85	0.030	2.50	-
Aromatic C	90	0.070	3.55	-
Aromatic H	91	0.030	2.42	-
Alcohol O	96	0.170	3.12	-
Alcohol H	97	0.000	0.00	-
Epoxy/Ether O	122	0.140	2.90	-
Primary Amine N	730	0.170	3.30	-
Secondary Amine N	731	0.170	3.30	-
Tertiary Amine N	732	0.170	3.30	-
Primary Amine H	739	0.000	0.00	-
Secondary Amine H	740	0.000	0.00	-
Fe (Octahedral)	-	$9.0298e^{-6}$	4.072	+1.575
Fe (Tetrahedral)	-	$9.0298e^{-6}$	4.072	+1.3125
O (Iron Oxide)	-	0.1554	3.166	-1.05
O (Hydroxyl Ion)	-	0.1554	3.166	-0.95
H (Hydroxyl Ion)	-	0.00	0.00	+0.425

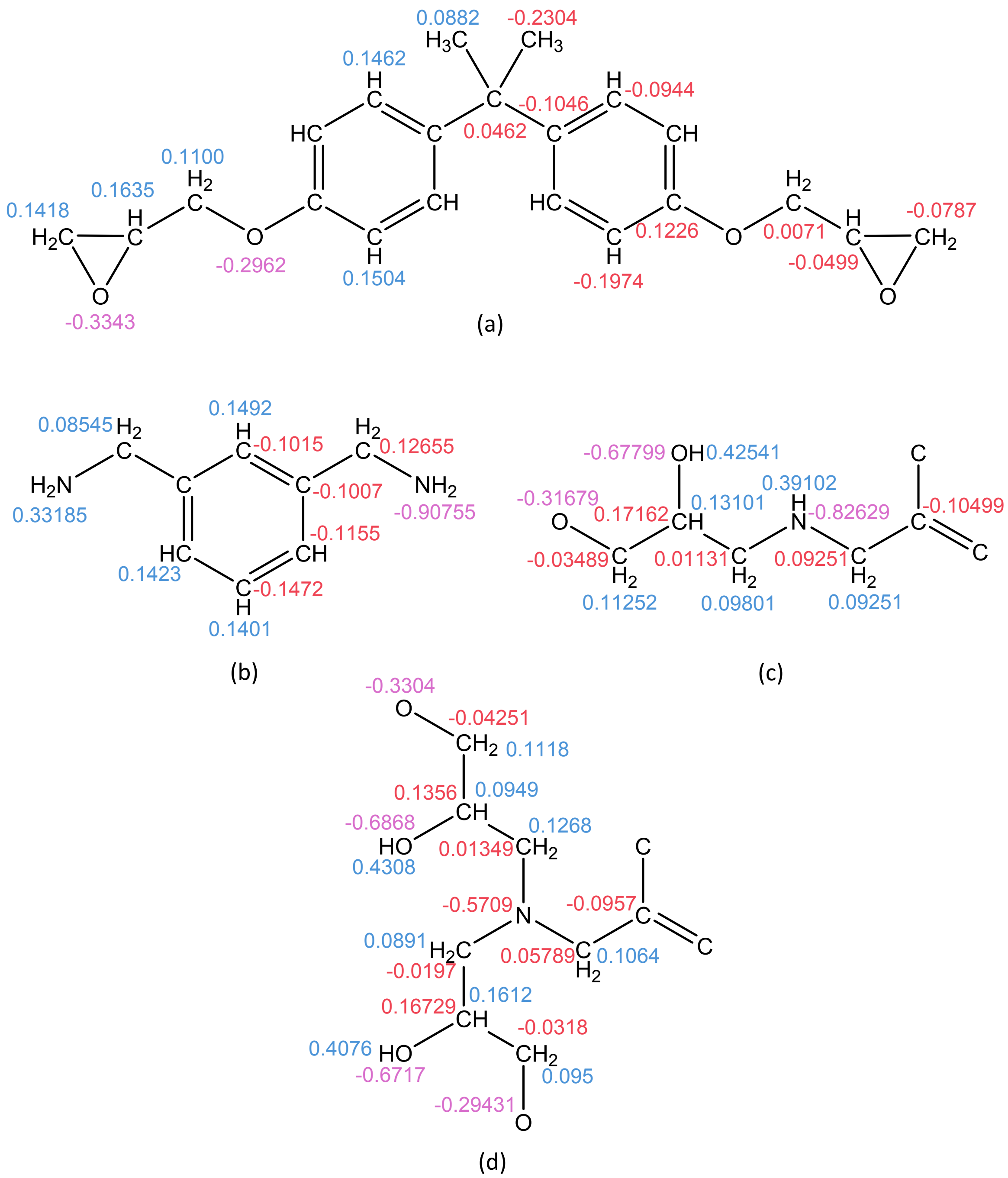

Figure S2. Skeletal structures of (a) DGEBA, (b) MXDA, (c) secondary amine fragment and (d) tertiary amine fragment. Atom charges are indicated beside corresponding carbon (red), hydrogen (blue) and heteroatom (purple) atoms. Atoms in equivalent positions with equal charges are excluded for simplicity.

Simulation Conditions

The first stage initialises the velocities of the monomers and mixes them, followed by compressing the initially disperse system to the density expected at 1 atm, using constant pressure (NPT) simulation. Prior to the introduction of the surface, harmonic force walls are in place at the top and bottom of the simulation box to maintain a 2D film structure of the monomer mixture. The

monomer mixing methodology prior to surface introduction and the cooling phase after crosslinking are adapted from the work of Demir and Walsh.[DOI: 10.1039/C5SM02788H]

Table S2. Simulation procedure.

Ensemble	Time / ns	Temperature / K
NVT	0.45	1000
NVT	0.5	1000-500
NVT	0.1	500
NPT	0.5	500
NVT	0.2	500
NVT	0.2	500-1000
NVT	1.0	1000
NVT	0.5	1000-500
NVT	0.2	500
NVT	0.2	500-1000
NVT	1.0	1000
Surface introduction		
NVT	1.0	1000-323
NVT	0.5	323
Crosslinking initiated		
NVT	7.0	323
NVT	0.5	323-500
NVT	7.5	500
Crosslinking halted		
NVT	3.0	600
NVT	15.0	600-300
NVT	1.5	300

Linear Concentration Plots

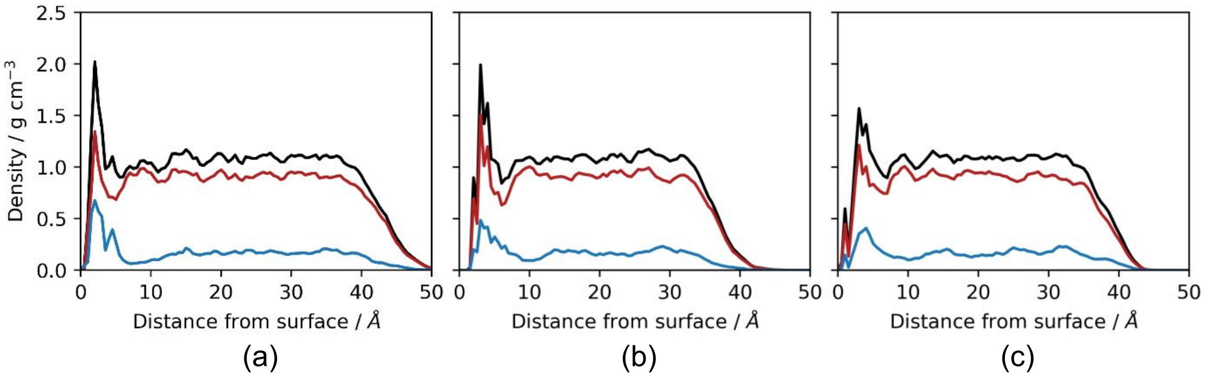

Figure S3. Linear density plots of (black) the entire crosslinked film, (red) DGEBA molecules and (blue) MXDA molecules. Plots are calculated for a film cured in contact with (a) hematite, (b) magnetite and (c) goethite.

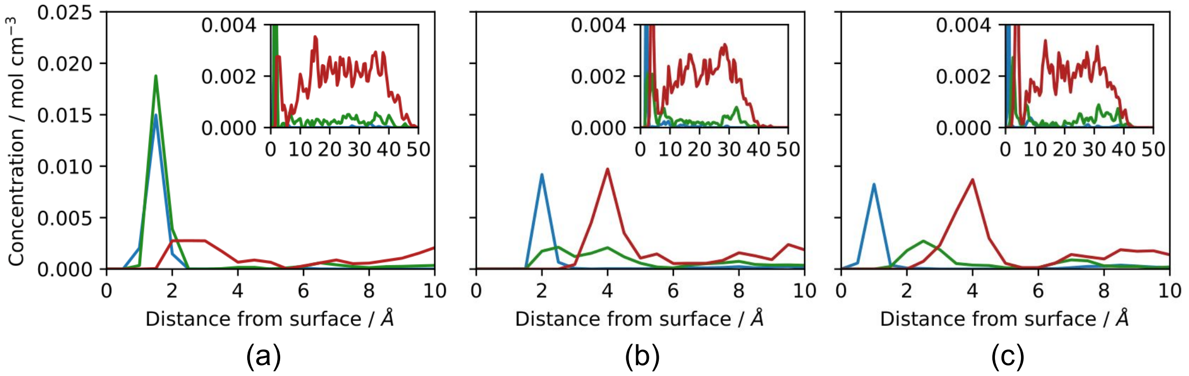

Figure S4. Linear concentration plots of (blue) primary, (green) secondary and (red) tertiary amine nitrogen atoms. Plots are calculated for a film cured in contact with (a) hematite, (b) magnetite and (c) goethite.

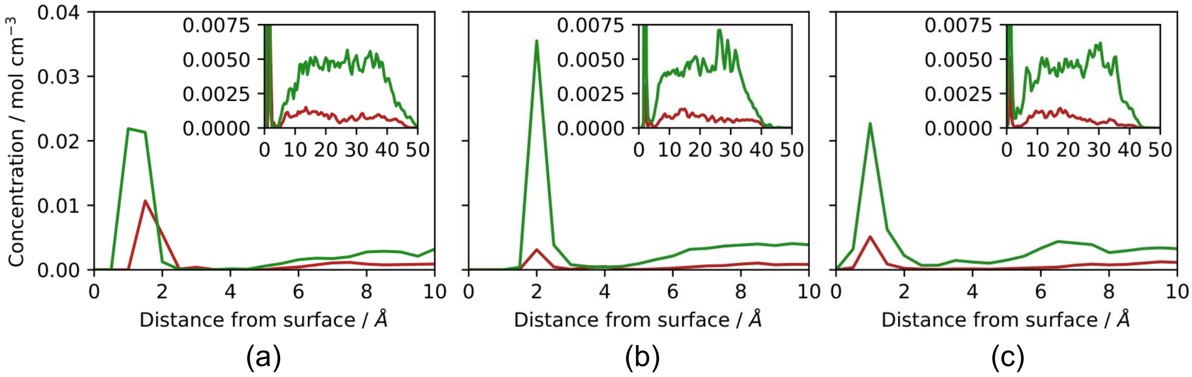

Figure S5. Linear concentration plots of (red) unreacted epoxy oxygen and (green) reacted alcohol/epoxy product oxygen atoms. Plots are calculated for a film cured in contact with (a) hematite, (b) magnetite and (c) goethite.

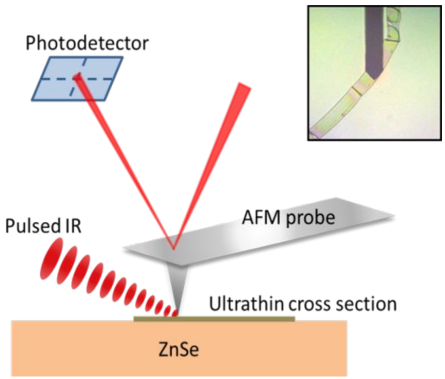

Scheme S1. The AFM-IR setup. Ultrathin cross-sections of DGEBA-MXDA iron oxide composites deposited onto a ZnSe support are scanned in contact mode whilst illuminated using a tuneable pulsed IR source. During the scan, deflection of the probe in response to IR pulses and sample height are monitored using a positioning laser reflected into the photodetector.
